# Editorial: Wild Immunology—The Answers Are Out There

**DOI:** 10.3389/fimmu.2019.00126

**Published:** 2019-01-30

**Authors:** Andrew S. Flies, Gregory M. Woods

**Affiliations:** ^1^Menzies Institute for Medical Research, University of Tasmania, Hobart, TAS, Australia; ^2^School of Medicine, University of Tasmania, Hobart, TAS, Australia

**Keywords:** natural disease model, host-pathogen, bats, tasmanian devil, out-of-the-box, re-wild, IgE, comparative immunology

Laboratory mice have been the workhorse of immunology research. Well-characterized, inbred mice raised in controlled environments have fostered an understanding of precise immunological mechanisms. Experimental variation has been minimized, but results rarely translate into successful therapies for humans or other animals (1, 2). Simple experimental modifications such as co-housing a pet shop mouse with laboratory mice can alter the immunophenotype of standard mouse strains (3). Considering the limitations of traditional laboratory immunology, intrepid researchers have delved into the nascent field of wild immunology. This “Wild Immunology—the answers are out there” Research Topic has collected original research articles, perspectives, and hypotheses. Capitalizing on wild immunology will help steer future research toward productive translational outcomes and fill knowledge gaps left by the standard immunology models.

Taking laboratory mice out of the box and re-wilding them in an outdoor enclosure is a simple approach to building on the vast amount of laboratory mouse immunology available. A DNA metabarcoding approach was used to quantify variation in feeding habits in a semi-natural environment, rather than the traditional approach of eliminating variation in the laboratory Budischak et al. Dietary quality and infection with *Trichuris muris* affected immune parameters (e.g., IgG1, IL-13) in the re-wilded mice, but did not reduce parasite load. This study also found that mice on the low-protein diet spent more time foraging, which could result in increased exposure to pathogens. Future re-wilding studies that incorporate disease models and experimental treatments should produce steady progress toward translational outputs by addressing natural variation from the outset, rather than only in clinical trials.

The emergence of various “omics” techniques (e.g., RNA transcriptomics) has opened new research pathways for non-model organisms. Transcriptomics were used for wild wood mice (*Apodemus sylvaticus*) to show that both “immune” and “non-immune” genes can be strong predictors of parasite burden Babayan et al. In this case the balance of protective immunity and tissue homeostasis resulted in relatively stable parasite loads and immune profiles. A significant negative association was found between worm burden and cytokine receptors (IL-5RA, IL-17RA). By contrast there were no significant positive or negative associations with cytokines, highlighting potential problems of serological assays focused on soluble cytokines that don't account for receptor expression.

The above two original studies using wild and re-wilded rodents were complemented by a thorough review of wild rodent immunological studies Viney and Riley. This immunology of wild rodents review is an excellent starting point for any researcher looking to venture into wild immunology. It shows how the study of wild rodents in a natural environment helps understand how immune responses contribute to fitness in the wild.

The prevalence of allergy is increasing in industrialized nations and is associated with dysregulated IgE effector functions. Hellman et al. found that dogs and Scandinavian wolves (*Canis lupus*) have serum IgE concentrations up to 200 times higher than non-allergic humans and laboratory rodents. Similar patterns have been observed in other wild species. A primary function of IgE is to induce degranulation of mast cells and basophils that rapidly amplify inflammatory responses upon initial infection by parasites Hellman et al. The reduced exposure to parasites in industrialized nations and the striking differences in IgE levels across species and environments suggests that immunological investigations of animals in natural settings could shed light on the growing allergy problem in humans. Importantly, Hellman et al. also provide thorough analysis and review of Fc receptors, which are necessary for most antibody-mediated effector functions, but are often overlooked in antibody-focused studies.

In addition to the rise in urban-associated diseases in humans, pathogens from wild animal populations can spillover into human and domestic animals. Bats (*Chiroptera*) are the second largest order of mammals and are host to a plethora of pathogens with the potential for cross-species transmission. A thought-provoking Hypothesis and Theory article describing unique aspects of the bat immune system proposed that two key features of the bat immune system might account for their ability to harbor pathogenic viruses Schountz et al. First, the IFN locus of the Australian black flying fox (*Pteropus alecto*) has fewer type I IFN genes than other mammals examined at that time, but that antiviral IFN-α mRNA is constitutively expressed at high levels (4). Second, the diversity of naive antibody repertoires in bats exceeds the diversity in other species studied to date [Schountz et al.; (5)]. The authors hypothesize that the combined “always on” IFN-α and diverse antibody repertoire allows bats to control infections at early stages, and thus minimize pathogenicity from virus and immunopathology Schountz et al. The long-life span of many bat species presents an opportunity to investigate changes in immunity across life history, which is limited in short-lived laboratory rodents.

Longitudinal studies of natural disease models can also fill gaps in our understanding of immunity and host-pathogen coevolution. A novel *Mycoplasma gallisepticum* pathogen emerged in wild house finches (*Haemorhous mexicanus*) and led to a 60% decrease in the wild finch population Vinkler et al. Comparison of pathogenicity of the original (1994) and more recent (2006) strains revealed that the increased virulence observed across this time scale has been driven in part by the host's pro-inflammatory response and incomplete host immunity [Vinkler et al.; (6)]. A similar pattern occurred with the myxoma virus used for rabbit (*Oryctolagus cuniculus*) control that began in the 1950s. An evolved 1990 myxoma virus strain became highly virulent because it induced septic shock like inflammatory response that led to immune collapse (7).

Simple and cost-effective disease surveillance tools and immunological reagents adaptable for a range of species are required to address issues of pathogen spillover, host-pathogen evolution, and immunity in natural settings. Glidden et al. assessed non-specific markers of inflammation in response to foot-and-mouth disease virus in African buffalo (*Syncerus caffer*). The buffalo were monitored for non-specific markers of inflammation every 2–3 months over 2 years. Serum haptoglobin was found to be a reliable surveillance marker of recent infection.

A cost-effective version of RNAseq (TagSeq) was used to fill the species-specific reagent void in a natural host-parasite system of threespine sticklebacks (*Gasterosteus aculeatus*) and native cestode parasites (*Schistocephalus solidus*) Lohman et al. Previous studies have shown that two geographically-isolated populations of sticklebacks are equally susceptible to infection, but that one population can suppress parasite growth substantially more than the other (8). The TagSeq analysis suggests that the parasite-resistant population had a dynamic gene expression profile, whereas the less-resistant population has a more static gene expression profile.

Wild immunology studies have value in understanding the evolution of the immune system. A review on melanomacrophage centers (MMC) highlighted that these aggregates of pigmented phagocytes can be found in vertebrates Steinel and Bolnick. As MMC share similarities with mammalian germinal centers it is tantalizing to suggest that MMC in fish have an immunological role and could be the evolutionary precursors of germinal centers. However, the authors caution that further genetic and functional studies are required before solid conclusions can be drawn.

The Tasmanian devil facial tumor disease (DFTD) has provided a rare opportunity to scrutinize the interplay between cancer and the immune system on a large scale in a natural tumor model. A vaccinate-and-release study of Tasmanian devils demonstrated that tumor-specific antibodies can be generated and represents a step forward in the search for a prophylactic vaccines Pye et al. This is a direct demonstration that wild immunology studies can have direct application to conservation and emerging disease solutions. The true test to evaluate any vaccine approach must occur in the wild, or in real-world contexts.

As functional experimental studies require significant resource investment (e.g., labor, reagents), using *in silico* analysis prior to embarking on wet lab studies and reagent development can yield more efficient use of limited resources. A comparative analysis of immune checkpoint molecules in two marsupials (*metatheria*) and seven placental species (*eutherians*) Flies et al. revealed that many key motifs and binding domains are conserved across more than 160 million years of evolution. The strong selective pressure to maintain immune checkpoints across species and environments suggests these checkpoints could offer stable therapeutic and vaccine targets for human disease and DFTD.

In summary, wildlife studies, particularly long-term monitoring studies, have begun addressing long-standing questions in immunology. Taking animals out of the lab and into more natural environments has yielded surprising results that are likely to help fill major knowledge gaps regarding the evolution and development of immunity in a wide range of species. We have only scratched the surface of the potential for wild immunology and look forward to finding more answers in the wild in the coming decades.

## Author Contributions

All authors listed have made a substantial, direct and intellectual contribution to the work, and approved it for publication.

### Conflict of Interest Statement

The authors declare that the research was conducted in the absence of any commercial or financial relationships that could be construed as a potential conflict of interest.
